# Bone health in a U.K. cohort of youth living with perinatally acquired HIV‐1: a longitudinal study

**DOI:** 10.1002/jia2.70029

**Published:** 2025-09-04

**Authors:** Merle Henderson, Alexandra Blenkinsop, Oliver Ratmann, Moira Cheung, Hermione Lyall, Sarah Fidler, Caroline Foster, the BONDY study group

**Affiliations:** ^1^ Department of Infectious Diseases Imperial College London London UK; ^2^ 900 Clinic Imperial College Healthcare NHS Trust London UK; ^3^ Department of Mathematics Imperial College London London UK; ^4^ Department of Paediatrics Great Ormond Street Hospital for Children London UK; ^5^ Department of Paediatrics Imperial College London London UK

**Keywords:** antiretroviral therapy, bone health, bone mineral density, bone mass, perinatal HIV, vitamin D

## Abstract

**Introduction:**

Low bone mineral density (BMD) has been described in children and young people with perinatally acquired HIV (PHIV), which may be related to both traditional (e.g. low body mass index and malnutrition) and HIV‐related risk factors (e.g. longstanding exposure to HIV and antiretroviral therapy [ART], with immune suppression, chronic immune activation and inflammation). Here, we evaluate BMD in a U.K. cohort of young people with PHIV by age and ART.

**Methods:**

This longitudinal, observational study was conducted at a U.K. tertiary PHIV service between November 2018 and March 2022. Bone health was assessed in 130 individuals aged 15–19 (*n* = 50), 20−24 (*n* = 50) and 25 years and older (*n* = 30) by dual‐energy X‐ray absorptiometry, bone mineralization and turnover markers. Low BMD was defined as lumbar spine (LS) and/or femur‐BMD z‐score below −2, relative to age, sex and ethnicity‐matched U.K. population‐based normative controls. Two‐year follow‐up evaluation was performed in those aged 15−19 (*n* = 42) and 20−24 years (*n* = 43) at enrolment, which included a group who switched from tenofovir disoproxil fumarate (TDF) to tenofovir alafenamide (TAF) ART at baseline. Bayesian logistic regression models examined predictors of low BMD and the effect of ART‐backbone on BMD accrual.

**Results:**

At baseline, 57% were female and 82% of black ethnicity, with 31 (24%) on TDF‐ART. Sixteen (12%) had low baseline BMD. Over a median follow‐up duration of 26 (interquartile range [IQR] 25–29) months, BMD accrual was lower‐than‐expected in those aged 15−19 years (mean change LS‐BMD z‐score −0.15 (standard deviation [SD] 0.44)), when compared to normative controls. No associations were seen with HIV parameters or the ART regimen. Participants who switched to TAF‐ART had similar BMD accrual 26 (IQR 24–32) months post switch, when compared to those on non‐TAF/TDF‐ART (mean change LS‐BMD z‐score TAF −0.01 [SD 0.41] vs. non‐TAF/TDF −0.03 [SD 0.54]).

**Conclusions:**

While rates of low BMD were reassuringly low in this cohort, lower‐than‐expected BMD accrual was observed in younger individuals, relative to normative controls. Overall, BMD accrual on TAF‐ART was non‐inferior to non‐TAF/TDF‐ART.

## 1 INTRODUCTION

Despite effective antiretroviral therapy (ART), multi‐system complications of chronic HIV are increasingly recognized in individuals with perinatally acquired HIV (PHIV) [1, 2]. Adverse bone health is of particular concern in young people as over half of adult bone mass accrual occurs during puberty, a period of rapid bone growth, which, if impaired, may result in a low adult peak bone mass (PBM) [3, 4]. Adult PBM is typically attained around 25 years of age, with low adult PBM an important determinant for the development of osteoporosis and resultant fracture risk in later life [3, 4, 5].

Low bone mineral density (BMD) has been described in children and adolescents with PHIV, defined as a dual‐energy X‐ray absorptiometry (DXA) z‐score of −2 or lower, with prevalence estimates ranging from 4% in high‐income countries [6], to 32% in low‐ and middle‐income countries [7, 8, 9, 10, 11]. The aetiology of low BMD in this population is likely multifactorial, related to both traditional (e.g. physical inactivity, malnutrition, low body mass index [BMI] and vitamin D deficiency) and HIV‐related putative risk factors (e.g. immunosuppression, uncontrolled viral replication and chronic inflammation). Initiation of ART has also been associated with a small decline in BMD, irrespective of regimen, and may be exacerbated by tenofovir disoproxil fumarate (TDF); a nucleotide reverse transcriptase inhibitor (NRTI) known to impact bone mineralization [3, 12]. These factors may negatively impact upon bone turnover, with a previous study in adolescents with PHIV demonstrating a correlation between low BMD and increased concentrations of bone turnover markers [7].

TDF is a commonly used, well‐tolerated NRTI, which has been licenced for use in children from 2 years of age since 2012 [13]. “TLD,” a generic co‐formulation of TDF, lamivudine and dolutegravir, is recommended by the World Health Organization as a first‐line ART regimen for those over 30 kg, including adolescents from 10 years of age [14]. Accordingly, TDF exposure during adolescence is now widespread. However, TDF use carries the risk of both nephrotoxicity and reduced BMD [13, 15, 16]. Tenofovir alafenamide (TAF), a prodrug of TDF with higher intracellular concentrations of the pharmacologically active metabolite tenofovir [14], is now recommended as an alternative NRTI in young people below 25 years of age, prior to the attainment of PBM, due to its improved renal and bone safety profile [17, 18, 19].

Despite these recommendations, limited data are available for the use of TAF in young people with PHIV [20, 21], or for the use of TDF in the emerging population of adults with PHIV aged 25 years and above, who have reached adult PBM. As increasing numbers of young people enter adult care, this study aimed to gain further data on the effect of age and ART use on BMD. We conducted a longitudinal, observational study in a U.K. cohort of young people with PHIV aged 15 years and over to evaluate markers of bone health and determine BMD, using DXA at baseline and after 24 months. Changes in bone health were compared by age group and ART regimen, including a cohort switching from TDF to TAF during the study period, associated with changes in national guidance. We hypothesized that BMD would be lower in PHIV, when compared to age‐matched normative data, with a normal rate of BMD accrual for those below 25 years of age on a non‐TDF‐containing ART regimen over a 2‐year follow‐up period.

## 2 METHODS

### 2.1 Study design and participants

This longitudinal, prospective observational study evaluated bone health in young people with PHIV attending a specialist HIV service at Imperial College Healthcare NHS Trust, London (U.K.) between November 2018 and March 2022. Individuals aged 15 years and older were enrolled into three cohorts based on age at study entry: 15−19, 20−24, and 25 years and older. Exclusion criteria included those with a weight of less than 35 kg, concurrent pregnancy or malignancy. Bone health was assessed by bone biochemistry and turnover markers, and DXA with vertebral fracture assessment. A 2‐year follow‐up evaluation was performed in those aged less than 25 years at enrolment, who had yet to achieve PBM accrual. All participants provided written informed consent prior to study enrolment. Ethics approval was gained from the London‐Stanmore Research Ethics Committee (REC) (18/LO/1474).

### 2.2 Study procedures

#### 2.2.1 Demographics and clinical evaluation

Baseline demographics, lifestyle and HIV‐related characteristics were recorded. Demographic characteristics included age, sex at birth, weight, height and ethnicity. BMI was calculated in kilograms (kg) divided by height in metres squared (kg/m^2^). BMI was converted into an age‐adjusted z‐score for those aged 15−19 years [22]. Lifestyle characteristics included the use of recreational drugs and/or alcohol, personal and family history of bone disease and reduced mobilization. Bone disease included a prior low‐impact fracture or other known bone condition. Reduced mobilization was defined as hypertonic diplegia and/or use of walking aids. HIV‐related characteristics included current and previous AIDS‐defining illnesses and CD4 counts, current plasma HIV RNA concentration, length of viral suppression, and current and historic ART regimens.

#### 2.2.2 Bone biochemistry and turnover markers

Bloods were collected for bone chemistry, including 25‐hydroxyvitamin D, parathyroid hormone (PTH) and bone profile (corrected calcium and phosphate). Bone turnover markers included procollagen type 1 N‐terminal propeptide (P1NP) (blood) and N‐terminal telopeptide (NTX) (urine), which were collected non‐fasting at a similar time of day (early afternoon). Samples were processed in‐house at NHS UKAS‐accredited laboratories using the following assays: Abbott Alinity I intact PTH and 25‐hydroxyvitamin D, Abbott Alinity C calcium, ZEUS ELISA NTX and Roche Cobas E411Elecsys Total P1NP. All samples were processed and analysed fresh, with the exception of NTX and P1NP, which were frozen at −20°C and analysed within 1 week. 25‐hydroxyvitamin D concentrations were considered abnormally low (insufficient) if ≤50 nmol/l, and PTH was raised if ≥7.2 pmol/l. These definitions were based on U.K. national guidance from the National Institute of Health and Care Excellence (NICE) and local NHS laboratory reference ranges [23].

#### 2.2.3 BMD markers

BMD was determined by DXA scan, which was taken of the full body, lumbar spine (LS) and both femurs. DXA scans were performed by trained radiographers, acquired on Lunar Prodigy or iDXA (General Electric (GE) Healthcare) scanners at St Marys Hospital, Imperial College Healthcare NHS Trust, according to local scanning protocols. Daily quality assurance calibrations were performed using a manufacturer‐provided phantom calibrated to the scanner, which were compared against previous calibration results and monitored over time. The precision error of DXA measurements was the root‐mean‐square standard deviation of the total body 0.010 g/cm^2^, AP spine L2−L4 0.010 g/cm^2^ and dual femur total 0.010 g/cm^2^. As this study utilized two different DXA scanners, a cross‐calibration was performed between scanners at the time of installation by a GE engineer to allow an accurate comparison of scans acquired across both scanners. Z‐scores were generated using U.K. population‐based normative controls: GE Healthcare AP spine and femur reference populations, Lunar enCORE software v18, matched for age, gender and ethnicity. The software used for analysis was GE Healthcare's Lunar enCORE platform (v18). Low BMD (below the expected range for age) was described as an LS (L2−4) and/or femur z‐score of below −2, as per the International Society for Clinical Densitometry guidelines [24, 25]. All participants with a baseline BMD z‐score below −1 to −2 at the LS and/or femur were referred to a dietician and given a leaflet promoting bone health. Those with a BMD z‐score below −2 were additionally referred to endocrinology. All individuals with abnormally low vitamin D were offered supplementation of colecalciferol, in line with national treatment guidelines.

#### 2.2.4 Follow‐up evaluation

A 2‐year follow‐up evaluation of all baseline study procedures, including bone biochemistry and turnover markers, as well as DXA scan, was performed in individuals aged 15−19 and 20−24 years at enrolment, who agreed to remain in the study.

### 2.3 Statistical analysis

Continuous variables were described by mean and standard deviation (SD) for normally distributed variables or median and interquartile range (IQR) for skewed variables. Categorical variables were described by counts and percentages.

The baseline binary outcome of low BMD was defined as an LS and/or femur BMD z‐score below −2. We first fitted Bayesian logistic regression models to examine the association of each of the putative risk factors with low LS and/or femur BMD, reporting posterior median odds ratios (OR) for each covariate with 95% credible intervals (CrI). As z‐scores were compared to age, sex and ethnicity‐matched normative population‐based data, no adjustment for confounders was implemented. We reported the posterior probability (PP) that the true ORs are larger than 1, given the data, which indicates the strength of evidence for an association (i.e. the risk factor is associated with an increased odds of low BMD). If the PP was close to 1, this suggests strong evidence that the risk factor increased the risk of low BMD (OR > 1); if the PP was close to 0, it indicates weak to no evidence that the risk factor increased the risk of low BMD. At follow‐up, we defined a binary outcome of any LS‐BMD z‐score below −2 as low BMD, as femur BMD z‐scores were not available in the 15−19 years age group. We computed the change in LS‐BMD z‐score and characterized the average change overall and by age group. We then fitted separate Bayesian linear regression models to the data, with each putative risk factor as a predictor, adjusting for baseline BMD z‐score. Factors associated with below‐average BMD accrual, when compared to population‐based normative data, were considered significant if the PP of the coefficient, corresponding to the expected change in BMD z‐score of <0, was close to 1. We further explored changes in bone health and turnover markers by computing the within‐person change in each marker between baseline and follow‐up. To estimate whether these changes were significant, we fitted a series of Bayesian hierarchical models with fixed effects for sex and random intercepts for each age group and identified covariates in which the PP of an increase (>0) or decrease (<0) in mean change was close to 1 (PP close to 1 considered statistically significant). Lastly, a Bayesian regression model was used to estimate the effect of TAF on BMD accrual relative to normative controls. This was performed by including TAF versus non‐TAF/non‐TDF‐ART regimens as a predictor and the duration on TAF in months, adjusting for baseline BMD z‐score. As empirical plots suggested the association between change in BMD z‐score and duration on TAF may be non‐linear, we modelled this relationship with a non‐parametric random function using a computationally efficient Hilbert‐Space Gaussian Process approximation [26]. All analyses were performed using R version 4.1.2., brms [27], CmdStan v2.34.1 [28], data.table v1.14.6 [29], readxl v1.4.1 [30], cmdstanr v0.7.1 [31] and ggplot2 v3.5.0 [32]. Further details on statistical methods, including the code used, are found in Supplementary Materials (Supplementary Methods).

## 3 RESULTS

### 3.1 Baseline characteristics

A total of 130 individuals were enrolled between November 2018 and December 2019. Fifty participants were recruited into each of the 15−19 and 20−24 year age groups, and 30 were aged 25 years and older. Of 130 individuals, 74 (57%) were female, 107 (82%) were of black ethnicity and with a median age of 21 (IQR 18–24) years. The median absolute CD4 count was 707 (486−926) cells/µl, and 89 (68%) had plasma HIV RNA concentrations <20 copies/ml, with 111 (85%) <200 copies/ml. Almost half of the cohort (64/130; 49%) had a previous history of an AIDS‐defining diagnosis (CDC Stage C). The most commonly prescribed ART regimen contained an integrase strand transfer inhibitor (INSTI) (45%), alongside an NRTI backbone, with 30 (24%) using TDF. Baseline characteristics are summarized in Table 1.

**Table 1:** Baseline cohort characteristics stratified by age group

Characteristic	Total cohort, *n* = 130	15−19 years *n* = 50	20−24 years *n* = 50	25+ years *n* = 30
**Demographics**				
Female sex at birth^a^	74 (57)	31 (62)	27 (54)	16 (53)
Ethnicity^a^				
Black African/Caribbean/British	107 (82)	42 (84)	40 (80)	25 (83)
Mixed/multiple	14 (11)	6 (12)	6 (12)	2 (2)
Asian/Asian British	3 (2)	2 (4)	1 (2)	0 (0)
Other	3 (2)	0 (0)	2 (4)	1 (3)
White	3 (2)	0 (0)	1 (2)	2 (7)
Born outside U.K.	78 (60)	29 (58)	30 (60)	19 (63)
Weight (kg)^b^	66.2 (14.6)	66.6 (7.1)	64.2 (13.0)	69.1 (12.1)
Height (cm)^b^	164.7 (8.7)	162.4 (7.8)	165.5 (9.8)	167.1 (7.5)
BMI (kg/m^2^)^b^	24.7 (4.8)	25.3 (4.9)	23.8 (4.9)	24.9 (3.8)
BMI z‐score^c^	N/A	0.83 (0.05−1.39)	N/A	N/A
Tanner stage				
IV	N/A	4 (8)	N/A	N/A
V	N/A	46 (92)	N/A	N/A
**HIV characteristics**				
Current CD4 (cells/µl)^c^	708 (486−926)	791 (508−927)	685 (427−799)	804 (557−989)
Nadir CD4 (cells/µl)^c^	249 (83−424)	369 (163−590)	210 (66−330)	230 (27−300)
Plasma viral load (copies/ml)^a^				
<20	89 (68)	35 (70)	32 (64)	22 (73)
20−200	22 (17)	8 (16)	7 (14)	7 (23)
>200	19 (15)	7 (14)	11 (22)	1 (3)
Previous CDC‐C diagnosis^a^	64 (49)	23 (46)	26 (52)	15 (50)
**ART characteristics** ^a^				
INSTI‐containing	59 (45)	27 (54)	20 (40)	12 (40)
PI‐containing	53 (41)	15 (30)	23 (46)	15 (30)
NNRTI‐containing	26 (20)	12 (24)	8 (16)	6 (20)
NRTI‐containing		48 (96)	46 (92)	26 (87)
TDF	31 (24)	8 (16)	12 (24)	11 (37)
TAF	35 (27)	14 (28)	16 (32)	5 (17)
Non‐TDF, non‐TAF	53 (41)	26 (52)	15 (30)	10 (33)
Previous TDF exposure	83 (64)	25 (50)	35 (70)	22 (73)
**Bone health characteristics** ^c^				
25‐hydroxyvitamin D (nmol/l)	33.9 (21.6−47.2)	35.9 (23.7−43)	30 (19.6−51.6)	32.9 (22.2−69.9)
Parathyroid hormone (pmol/l)	6.0 (4.4−8.6)	4.9 (3.7−6.9)	6.6 (5.0−8.9)	6.4 (5.3−8.9)
Corrected calcium (mmol/l)	2.4 (2.4−2.5)	2.4 (2.4−2.5)	2.4 (2.3−2.4)	2.4 (2.3−2.4)
Phosphate (mmol/l)	1.1 (1.0−1.2)	1.2 (1.1−1.3)	1.1 (1.1−1.2)	1.1 (1.1−1.3)
Alkaline phosphate (IU/l)	82.0 (68.0−103.8)	91 (72.5−125.0)	81.0 (67.2−96.8)	71.5 (63.5−84.2)
NTX (BCE/mmol creatinine)	52.0 (34.0−75.8)	75.5 (55.0−104.5)	51.5 (39.2−65.8)	27.5 (18−35.2)
P1NP (µg/l)	93.2 (66.6−118.6)	117.1 (93.4−169.3)	89.8 (64.8−105.3)	61.9 (46.3−78.5)
**BMD DXA characteristics**				
LS‐BMD z‐score^b^	−0.43 (1.4)	−0.12 (1.33)	−0.83 (1.3)	−0.27 (1.57)
Femur‐BMD z‐score^b^	−0.55 (1.13)	N/A	−0.58 (1.09)	−0.51 (1.21)
LS/femur‐BMD z‐score below −2^a^	16 (12)	3 (6)	8 (16)	5 (17)

Abbreviations: ART, antiretroviral therapy; BCE, bone collagen equivalents; BMD, bone mineral density; BMI, body mass index; CDC‐C, Centres for Disease Control and Prevention—Category C (AIDS); DXA, dual X‐ray absorptiometry; INSTI, integrase strand transfer inhibitor; NNRTI, non‐nucleoside reverse transcriptase inhibitor; NRTI, nucleoside reverse transcriptase inhibitor; N/A, not applicable; NTX, N‐terminal telopeptide; P1NP, procollagen type 1 N‐terminal propeptide; PI, protease inhibitor; PTH, parathyroid hormone; TAF, tenofovir alafenamide; TDF, tenofovir disoproxil.

^a^ *n* (% of denominator).

^b^ Mean (SD).

^c^ Median (IQR).

### 3.2 Baseline bone assessment

**Figure 1:**
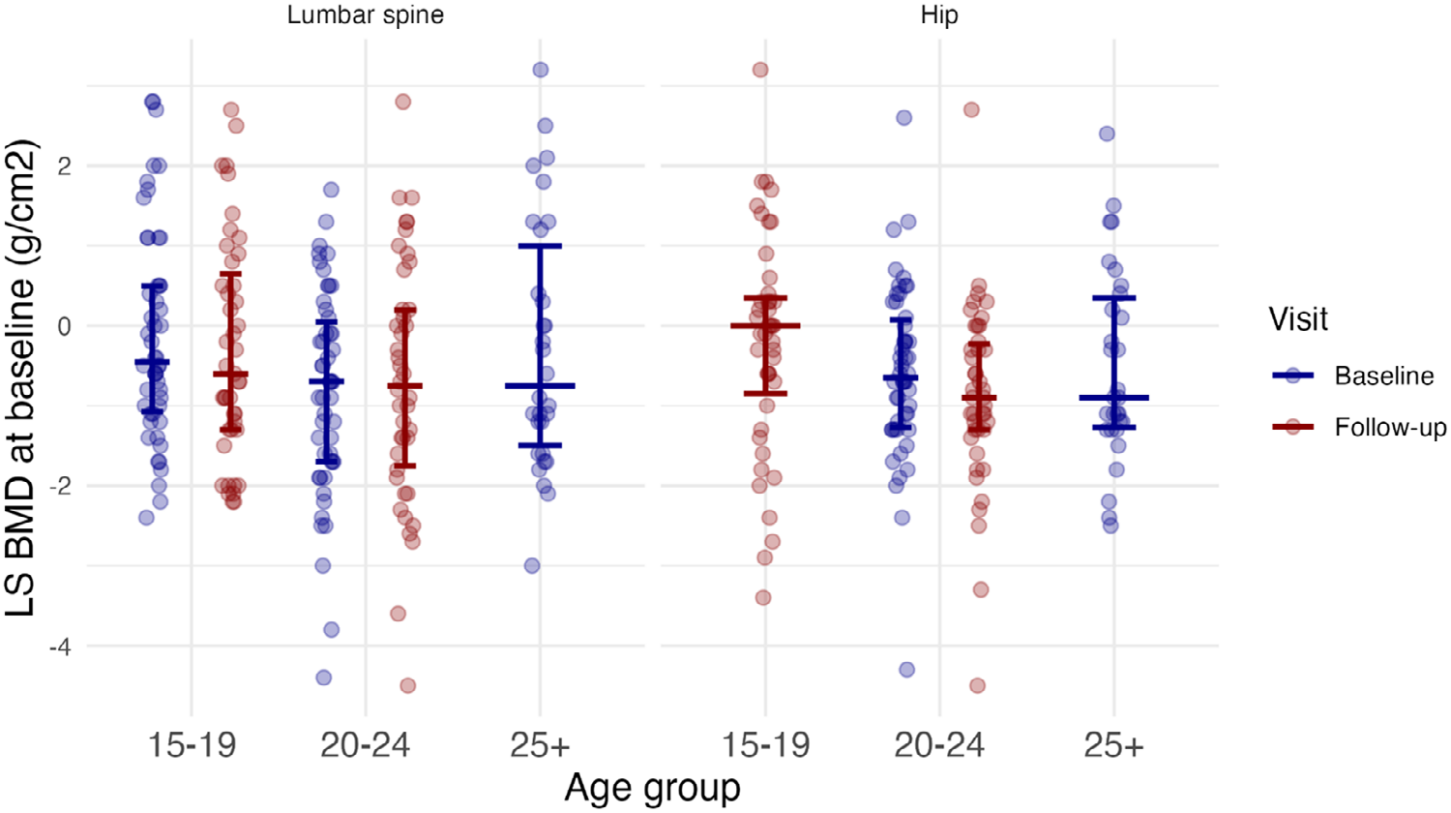
Bone mineral density (BMD) z‐score at baseline by age group, displayed with mean and standard deviation.

The mean BMD z‐score was −0.43 (SD 1.4) at the LS and −0.55 (SD 1.13) at the femur (Table 1). Sixteen (12%) had a low (less than −2) LS‐BMD z‐score; three (6%) aged 15−19 years, eight (16%) 20−24 years and five (17%) 25 years and over (Figure 1). A further 49 (38%) individuals were identified as at risk of low BMD with a LS and/or femur BMD z‐score below −1 to −2 (Table S1). 25‐hydroxyvitamin D concentrations were abnormally low (≤50 nmol/l) in 100 (77%) individuals, with a high PTH (≥7.2 pmol/l) in 47 (36%); by age group, low 25‐hydroxyvitamin D concentrations were 42 (32%), 37 (29%) and 21 (16%), and high PTH 12 (9%), 24 (18%) and 11 (9%), respectively. Bone turnover markers were highest in those aged 15−19 years, which declined with age (Table 1). In a univariate linear regression analysis, there were no factors significantly associated with an increased odds of low BMD (PP OR >1); however, a trend towards significance was seen with older age, reduced mobilization, lower BMI, current or previous smoking and duration on TDF (Table 2).

**Table 2:** Baseline associations of putative risk‐factors with low lumbar spine (LS) bone mineral density (BMD) z‐score below −2

Covariate	OR	95% CrI	Posterior probability OR >1^*^
**Traditional, non‐HIV‐related risk factors**			
Age (years)			
20−24 versus 15−19	1.88	0.69, 5.1	0.89
25+ versus 15−19	1.80	0.58, 5.43	0.88
Family history of bone disease	1.31	0.38, 4.13	0.69
Reduced mobilization	2.56	0.77, 7.85	0.94
BMI (kg/m^2^)			
<18.5 versus 18.5−25	2.03	0.35, 12.26	0.79
25−30 versus 18.5−25	0.62	0.19, 1.82	0.20
30+ versus 18.5−25	0.53	0.13, 1.92	0.17
Current smoker versus never/ex	1.87	0.63, 5.39	0.88
Drinks alcohol	1.35	0.51, 3.44	0.73
25‐hydroxyvitamin D ≤ 50 nmol/l	0.71	0.27, 1.97	0.25
PTH ≥ 7.2 pmol/l	0.80	0.30, 2.11	0.32
**HIV‐related risk factors**			
Prior CDC‐C/CD4 <200 cells/µl or <20%	1.1	0.42, 2.85	0.58
Prior TDF exposure	1.22	0.48, 3.32	0.65
Duration on TDF (years)	1.06	0.94, 1.18	0.85
ART regimen			
PI versus TDF	1.26	0.35, 4.24	0.64
INSTI versus TDF	0.77	0.23, 2.55	0.34
NNRTI versus TDF	0.44	0.08, 1.94	0.16

Abbreviations: ART, antiretroviral therapy; BMD, bone mineral density; BMI, body mass index; CDC‐C, Centres for Disease Control and Prevention—Category C (AIDS); CrI, credible interval; INSTI, integrase strand transfer inhibitor; NNRTI, non‐nucleoside reverse transcriptase inhibitor; NRTI, nucleoside reverse transcriptase inhibitor; OR, odds ratio; PI, protease inhibitor; PTH, parathyroid hormone; TDF, tenofovir disoproxil.

* Posterior probability OR >1 indicates covariate is associated with abnormal BMD.

### 3.3 Longitudinal assessment of BMD accrual

Of the 100 individuals aged less than 25 years at recruitment, and, therefore, eligible for visit 2, 85 attended for a longitudinal evaluation of bone health between April 2021 and March 2022. Of these 85 individuals, 43 were 15−19 years and 42 were 20−24 years at enrolment. Median follow‐up duration was 26 (IQR 25–29) months. Reasons for study withdrawal included: non‐attendance (5), transfer of care (4), psychosis (2), pregnancy (1), current AIDS diagnosis (1), loss to follow‐up (1) and study withdrawal (1).

At follow‐up, the mean BMD z‐score was −0.50 (SD 1.46) at the LS and −0.54 (SD 1.32) at the femur (Figure 1). Mean change in LS‐BMD z‐score from baseline was −0.02 (SD 0.51). Individuals with PHIV aged 15−19 years at enrolment had lower‐than‐expected BMD accrual, relative to normative age, sex and ethnicity‐matched population data (mean change in LS‐BMD z‐score −0.15 [SD 0.44]; a change of 0 indicating normal BMD accrual). This was in contrast to those aged 20−24 years who had a higher‐than‐expected rate of BMD accrual, 0.11 (SD 0.54), relative to matched data. 25‐hydroxyvitamin D concentrations increased significantly between baseline and follow up among those aged 15−19 and 20−24 years at enrolment (mean change 25‐hydroxyvitamin D 15–19 years 7.72 [95% CrI 2.29, 13.04] nmol/l and 20–24 years 7.74 [95% CrI 2.37, 13.15] nmol/l, both PP = 0.99; Table S3); however, 25‐hydroxyvitamin D concentrations below 50 nmol/l persisted in 51 (60%) individuals and high PTH in 24 (28%). P1NP decreased significantly between baseline and follow‐up among those aged 15–19 years only (mean change P1NP −44.33 [95% CrI 16.38, −8.6], PP = 0.99). In regression analyses, there were no clear associations of putative risk factors at baseline with lower‐than‐expected BMD accrual (Table 3).

**Table 3:** Associations of putative risk‐factors at baseline with low bone mineral density (BMD) accrual

Covariate	Estimated coefficient	95% CrI	Posterior probability coefficient <0^*^
**Traditional, non‐HIV‐related risk factors**			
Age (years)			
20−24 versus 15−19	−0.25	−0.03, 0.47	0.02
Family history of bone disease	−0.14	−0.19, 0.45	0.20
Reduced mobilization	−0.18	−0.51, 0.14	0.85
BMI (kg/m^2^)			
<18.5 versus 18.5−25	−0.10	−0.77, 0.77	0.52
25−30 versus 18.5−25	−0.07	−0.84, 0.74	0.57
30+ versus 18.5−25	−0.19	−1.01, 0.63	0.68
Current smoker versus never/ex	−0.24	−0.04, 0.54	0.05
Drinks alcohol	−0.06	−0.17, 0.30	0.31
25‐hydroxyvitamin D ≤ 50 nmol/l	−0.06	−0.22, 0.34	0.32
PTH ≥ 7.2 pmol/l	−0.80	−0.30, 2.11	0.01
**HIV‐related risk factors**			
Prior CDC‐C/CD4 <200 cells/µl or <20%	−0	−0.24, 0.24	0.48
Prior TDF exposure	−0.06	−0.17, 0.3	0.29
Duration on TDF (years)	−0.02	−0.01, 0.05	0.09
ART regimen			
PI versus TAF	−0.26	−0.6, 0.08	0.92
INSTI versus TAF	−0.21	−0.46, 0.03	0.96
NNRTI versus TAF	−0.27	−0.06, 0.6	0.05

Abbreviations: ART, antiretroviral therapy; BMD, bone mineral density; BMI, body mass index; CDC‐C, Centres for Disease Control and Prevention—Category C (AIDS); CrI, credible interval; INSTI, integrase strand transfer inhibitor; NNRTI, non‐nucleoside reverse transcriptase inhibitor; NRTI, nucleoside reverse transcriptase inhibitor; OR, odds ratio; PI, protease inhibitor; PTH, parathyroid hormone; TAF, tenofovir alafenamide; TDF, tenofovir disoproxil.

* Posterior probability coefficient <0 indicates covariate is associated with abnormal BMD accrual.

### 3.4 Impact of switching NRTI‐backbone on BMD accrual

**Figure 2:**
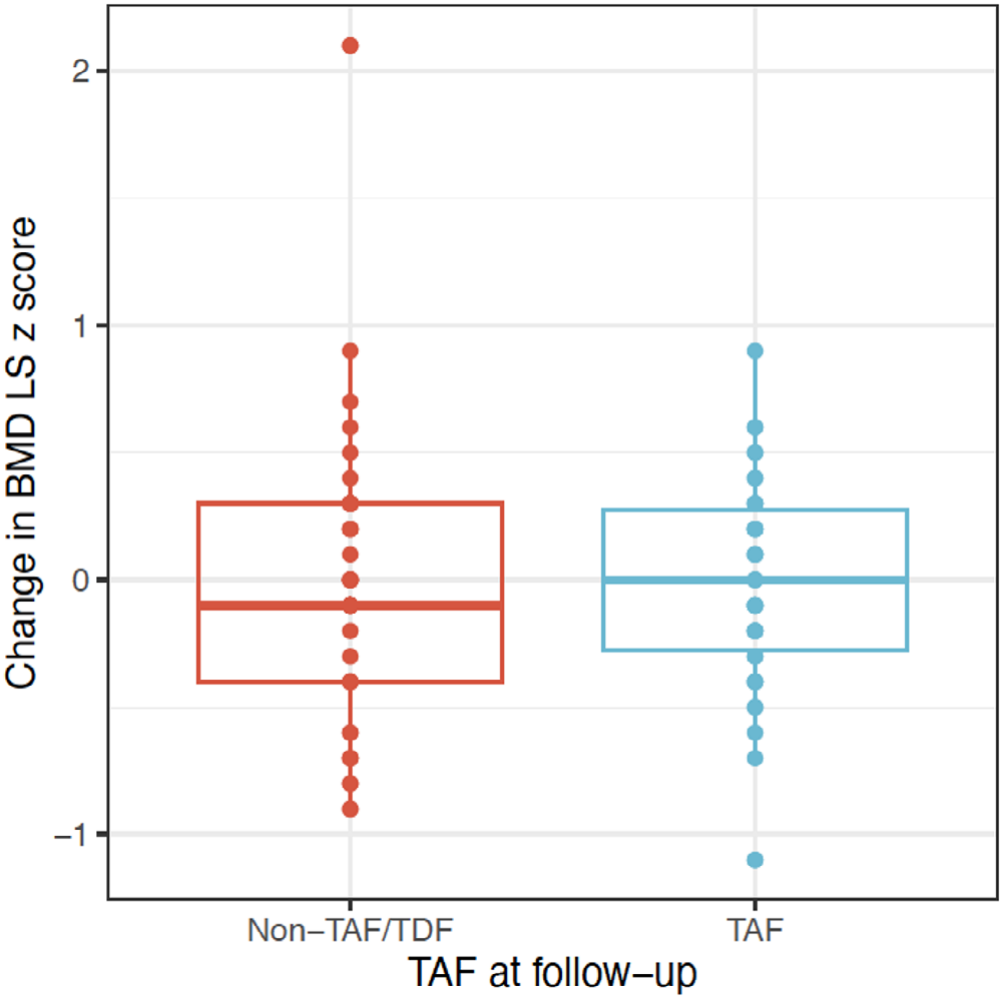
Change in lumbar spine (LS) bone mineral density (BMD) z‐score in those taking TAF versus non‐TDF/non‐TAF‐ART. Abbreviations: ART, antiretroviral therapy; TAF, tenofovir alafenamide; TDF, tenofovir disoproxil fumarate.

A sub‐analysis was performed to determine the effect of switching to a TAF‐containing ART regimen (TAF‐ART) on LS‐BMD z‐score, when compared to continuing other non‐TAF/non‐TDF‐containing ART regimens; dual NRTI backbone (abacavir/lamivudine), INSTI/non‐nucleoside reverse transcriptase inhibitor (NNRTI), boosted protease inhibitor (bPI)/INSTI or PI monotherapy. Despite a change in national guidelines, two participants chose to remain on a TDF‐containing ART regimen (TDF‐ART) and were excluded from further analyses. At follow‐up, 43 (51%) were on TAF‐ART, for a median duration of 26 (IQR 24–32) months. Over a 2‐year follow‐up period, participants who switched to TAF‐ART had similar BMD accrual to participants on non‐TAF/non‐TDF‐ART regimens (mean change LS‐BMD z‐score TAF −0.01 [SD 0.40] vs. non‐TAF/non‐TDF −0.03 [SD 0.56]) (Figure 2), with no significant effect of TAF on BMD accrual (PP coefficient <0 = 0.58) (Table S2).

## 4 DISCUSSION

This longitudinal cohort study assessed bone health in young people with PHIV aged 15 years and older. We report a low overall frequency of low BMD, with trends towards an association with both traditional (older age, reduced mobilization, lower BMI, current or previous smoking) and HIV‐related putative risk factors (duration on TDF). Interestingly, we found that the frequency of low BMD was higher in this U.K. cohort (12%), when compared to similar high‐income settings, such as within the United States (4%) and the Netherlands (8%) [6, 33]. Notably, those participants were younger and with a history of less severe HIV disease, when compared to our cohort; a high proportion of whom were of black ethnicity and born outside the U.K., which may have contributed to the observed differences.

A high proportion of our cohort were vitamin D insufficient, 85% of individuals at baseline, and 65% had hyperparathyroidism. This may be related to limited sunshine duration and exposure in the U.K., and the predominately black ethnicity of participants (82%), both known contributors towards low 25‐hydroxyvitamin D concentrations. Vitamin D deficiency is a recognized risk factor for low BMD, which can accelerate bone demineralization through hypocalcaemia and secondary hyperparathyroidism [34]. Madanhire and colleagues recently demonstrated an association between 25‐hydroxyvitamin D concentrations <75 nmol/l and increased PTH, as well as increased bone turnover markers in Black African adolescents with PHIV [35]. However, in sensitivity analyses stratified by 25‐hydroxyvitamin D concentrations <50 nmol/l versus ≥50 nmol/l, normal physiological feedback relationships between PTH, active vitamin D and bone turnover markers were only observed in those with concentrations ≥50 nmol/l. As highlighted by the authors, these findings may suggest that first, vitamin D insufficiency/deficiency may lead to a disruption in bone metabolic regulation and second, that higher 25‐hydroxyvitamin D concentrations above 75 nmol/l are required for optimal bone health in young people with PHIV in certain settings, such as Southern Africa [35]. Previous studies of young people with HIV have demonstrated an association between low 25‐hydroxyvitamin D concentrations and total‐body BMD [36, 37]. However, the effects of vitamin D supplementation on BMD in young people with PHIV have been conflicting; a recent randomized controlled trial in Thailand observed increases in BMD and a reduction in bone turnover markers in individuals with PHIV aged 10−19 years with vitamin D/calcium supplementation either at standard‐dose (400 IU/1200 mg/day) or high‐dose (400 IU/1200 mg/day plus ergocalciferol 20,000 IU/week), most pronounced in the high‐dose group [38]. Vitamin D supplementation was stopped at study completion and 3 years later, despite significant reductions in median serum 25‐hydroxyvitamin D concentration and an increase in PTH, no decline in LS‐BMD was reported [39]. Whether vitamin D supplementation during key periods of bone development has lasting effects on bone health is unknown [39], and further longitudinal follow‐up is warranted. However, these results are not consistent, and other studies have demonstrated no effect of vitamin D supplementation on bone mass accrual in children and adolescents with HIV [40, 41, 42]. These discrepancies may be related to such factors as variations in dose, formulation and duration of supplementation, as well as medication adherence and degree of vitamin D deficiency. In this study, as expected, we found that bone turnover markers NTX and P1NP were highest in the youngest age groups and declined with age. These findings are in keeping with previous studies, which demonstrated bone turnover markers to be highest during puberty, decreasing towards adult levels in late puberty [43, 44], suggesting that markers stabilize following cessation of bone growth and with normal maturation.

Over a 2‐year follow‐up period, change in BMD accrual in those aged 15−19 years at enrolment was lower than that of age, sex and ethnicity‐matched peers; findings which have been reflected in younger people with PHIV [45]. Data from a North American cohort of 172 children with PHIV aged 7−16 years demonstrated that younger children had slower BMD accrual, when compared to children without HIV, which nearly normalized at older ages but did not catch‐up with the BMD accrual achieved by those without HIV [45]. Unexpectedly, we found that those aged 20–24 years at enrolment had a higher rate of BMD accrual (LS‐z‐score 0.11 [SD.54]) relative to reference data, the cause of which is unclear, and may, in part, reflect variation in BMD accrual across this age group, or delayed skeletal maturation due to delayed in growth and pubertal maturation associated with chronic disease.

Reassuringly, the rate of BMD accrual for those below 25 years of age on TAF‐ART was non‐inferior to other non‐TAF/non‐TDF‐ART regimens over a 2‐year follow‐up period. These findings are in line with data from the recent CHAPAS‐4 study; a randomized control trial which compared TAF/emtricitabine versus current standard‐of‐care (abacavir or zidovudine with lamivudine) as NRTI‐backbone options for second‐line ART in African children aged 3–15 years [21]. No significant differences were identified in LS‐BMD z‐scores by treatment arm over a 96‐week period (DXA scans in 170 children at weeks 0, 48 and 96). Taken together, these data suggest a limited risk of bone toxicity related to the use of TAF‐ART. However, other non‐bone‐related toxicities have been demonstrated in adults with HIV on TAF‐ART, including weight gain and elevated cholesterol [46, 47, 48]; side effects also seen with INSTIs and bPIs, respectively [49, 50]. In our cohort, almost half were overweight (27%) or obese (14%), highlighting the need for caution in ART prescribing, balancing potential bone health advantages of TAF‐ART against the potential metabolic side effects.

There were several strengths of this study. First, a longitudinal evaluation of bone health was performed at baseline and after 2 years in a well‐characterized cohort of individuals with PHIV, with the long duration of follow‐up allowing for a more meaningful assessment of BMD accrual over time. Second, as previous research on bone health in those with PHIV has generally been limited to children and adolescents, inclusion of an older age‐group, over the age of 25 years, with a long cumulative exposure to ART, provides timely data on an under‐represented cohort in PHIV research. There were also limitations, such as the lack of a matched control group without HIV. While we were able to compare data on BMD z‐scores to normative, population‐based age, sex and ethnicity‐matched data, this population‐based reference database may not fully account for ethnic and geographic variability in bone density development [51]. We also acknowledge the potential for selection bias, as our cohort may be skewed towards individuals engaged in regular care, rather than those with inconsistent healthcare attendances or those out of care. Further, although our study included over half of the entire clinic cohort, the relatively small sample size may have impacted on our ability to determine associations with low BMD. Lastly, while the study cohort was drawn from a single, specialist centre in a high‐income setting, most of the study population was of Black ethnicity, which may be more reflective of the global population living with PHIV, improving the generalizability of these results.

## 5 CONCLUSIONS

In this cohort of young people with PHIV, there was a low prevalence of low BMD. Changes in BMD accrual over 2 years of follow‐up were lower‐than‐expected, when compared to age, sex and ethnicity‐matched U.K. population‐based normative data. No associations were seen with the severity of HIV disease or ART regimen. Reassuringly, the rate of BMD accrual on TAF‐ART was non‐inferior to other non‐TAF/non‐TDF‐ART regimens over a 2‐year follow‐up period.

## COMPETING INTERESTS

The authors have no conflicts of interest to declare in relation to this work.

## AUTHOR CONTRIBUTIONS

All authors reviewed and approved the final manuscript. MH contributed to data analysis and writing—manuscript preparation and editing. AB performed statistical analysis. OR provided statistical support. MC contributed to data interpretation. HL supported protocol implementation and recruitment. SF conceptualization, protocol implementation and recruitment, writing—review and editing, supervision. CF conceptualization, funding acquisition, protocol implementation, principal investigator, recruitment, writing—review and editing, supervision.

## FUNDING

The study was funded by Gilead investigator‐led award (Dr Caroline Foster).

## Supporting information




**Table S1**. Baseline associations of putative risk‐factors with lumbar spine (LS) bone mineral density (BMD) z‐score below −1.
**Table S2**. Association of TAF versus non‐TAF/TDF with bone mineral density (BMD) accrual.
**Table S3**. Changes to bone health markers over the study period.

## Data Availability

The data that support the findings of this study are available on request from the corresponding author. The data are not publicly available due to privacy or ethical restrictions.
