# Supplementary material for: Bone health in a U.K. cohort of youth living with perinatally acquired HIV‐1: a longitudinal study
Source: J Int AIDS Soc. 2025 Sep 4;28(9):e70029. doi: 10.1002/jia2.70029 (PMC12409145; doi:10.1002/jia2.70029)
Supplement: Supplementary file 1 — Table S1. Baseline associations of putative risk‐factors with lumbar spine (LS) bone mineral density (BMD) z‐score below −1. Table S2. Association of TAF versus non‐TAF/TDF with bone mineral density (BMD) accrual. Table S3. Changes to bone health markers over the study period. [file JIA2-28-e70029-s001.docx]

**Supplementary Material**

**S1 Supplementary Methods**

**S1.1 Risk factors for abnormal BMD**

To identify risk factors for abnormal BMD at baseline, we fitted independent Bayesian logistic regression models with weakly informative prior of the general form,

$\boldsymbol{Y\sim}\mathrm{Bernoulli}\boldsymbol{(\pi)}$ $(1)$

$$\mathrm{logit}\left( \boldsymbol{\pi} \right)=\alpha+\boldsymbol{\beta X}$$

$\alpha\sim N\left( {0,5}^{2} \right)$

$\boldsymbol{\beta}\sim N\left( 0,1 \right)$,

where $\boldsymbol{Y}$ is a binary outcome indicating abnormal BMD in lumbar spine and/or femur, $\alpha$ is the intercept term, $\boldsymbol{X}$ is an $n\times p$ design matrix of covariates and $\boldsymbol{\beta}$ is a vector of their corresponding model parameters.

Numerical inference was carried out with Stan using the brms package in R, across 4 chains of 2000 iterations each and a burn-in of 1000. Code is available at github.com/MLGlobalHealth/bondy.study. All models converged well with no observed divergences.

**S1.1 Risk factors for accrual ofBMD**

To identify risk factors for the accrual of BMD relative to matched controls, we fitted independent Bayesian linear regression models of the general form,

$\boldsymbol{Y}=\alpha+\boldsymbol{\beta X+}\gamma\boldsymbol{Z}$ $(2)$

$\alpha\sim N\left( {0,5}^{2} \right)$

$$\boldsymbol{\beta}\sim N\left( 0,1 \right)$$

$\gamma\sim N\left( 0,1 \right)$,

where $\boldsymbol{Y}$ is a continuous outcome corresponding to change in LS-BMD z-score between baseline and follow-up, $\boldsymbol{Z}$ are participant baseline z-scores and $\boldsymbol{\gamma}$ is the corresponding model parameter. $\alpha$, $\boldsymbol{X}$ and $\boldsymbol{\beta}$ are as before.

We estimated the effect of TAF on BMD accrual in a separate model which also adjusted for the duration on TAF in months, which we modelled as a non-linear random function approximated by a Hilbert-Space Gaussian Process. The full model is described by,

$$\boldsymbol{Y}= \alpha+\beta_{BL}\boldsymbol{x}_{BL}+ {(\beta}_{TAF} \boldsymbol{+}f(\boldsymbol{x}_{t}))\boldsymbol{x}_{TAF}\boldsymbol{)}(3)$$

$$f\left( \boldsymbol{x}_{t} \right)\sim HSGP\left( 0,k\left( x_{t},x_{t}^{'} \right) \right)$$

$$k\left( x_{t},x_{t}^{'} \right)=\sigma^{2}\exp\left( \frac{\left( x_{t}-x_{t}^{'} \right)}{2\mathcal{l}^{2}} \right)$$

$$\alpha\sim N\left( {0,5}^{2} \right)$$

$$\boldsymbol{\beta}\sim N\left( 0,1 \right)$$

$$\sigma\sim N\left( {0,0.5}^{2} \right)$$

$$\mathcal{l}\sim N\left( {0,0.2}^{2} \right),$$

where $\boldsymbol{x}_{BL}$ is the baseline BMD lumbar spine z-score $\boldsymbol{x}_{TAF}$F is an indicator for participants on TAF at follow-up, $\boldsymbol{x}_{t}$ is the duration spent on TAF, $k\left( x_{t},x_{t}^{'} \right)$ is the kernel function, for which we assume a squared exponential. $\sigma$ and $\mathcal{l}$ are the hyper-parameters of the kernel function, which determine the amplitude and smoothness of the random function. We approximate the GP with 30 basis functions and a factor of $c=1.5$ to compute the boundary value.

**S2 Supplementary Tables**

| **Supplementary table 1. Baseline associations of putative risk-factors with lumbar spine (LS) bone mineral density (BMD) z-score below -1** | | | |
| --- | --- | --- | --- |
| **Covariate** | **OR** | **95% CrI** | **Posterior probability OR >1*** |
| **Traditional, non-HIV-related risk factors** | | | |
| Age (years) |  |  |  |
| 20-24 vs. 15-19 | 1.91 | 0.95-3.99 | 0.96 |
| 25+ vs. 15-19 | 3.00 | 1.27-7.10 | 1.00 |
| Family history of bone disease | 2.05 | 0.82-5.25 | 0.93 |
| Reduced mobilisation | 2.69 | 0.91-8.15 | 0.96 |
| BMI (kg/m^2^) |  |  |  |
| <18.5 vs. 18.5-25 | 1.51 | 0.26-8.90 | 0.67 |
| 25-30 vs. 18.5-25 | 0.57 | 0.27-1.18 | 0.07 |
| 30+ vs. 18.5-25 | 0.34 | 0.12-0.91 | 0.02 |
| Current smoker vs. never/ex | 1.31 | 0.58-2.95 | 0.73 |
| Drinks alcohol | 1.19 | 0.61-2.34 | 0.69 |
| 25-hydroxyvitamin D ≤ 50 nmol/L | 0.45 | 0.21-0.97 | 0.02 |
| PTH ≥ 7.2 pmol/L | 0.74 | 0.38-1.46 | 0.20 |
| **HIV-related risk factors** |  |  |  |
| Prior CDC-C or CD4 <CD4 200 cells/ul or <20% | 1.35 | 0.69-2.64 | 0.81 |
| Prior TDF exposure | 1.45 | 0.73-2.92 | 0.86 |
| Duration on TDF (years) | 1.07 | 0.99-1.17 | 0.95 |
| ART-regimen |  |  |  |
| PI vs. TDF | 1.50 | 0.55-4.13 | 0.79 |
| INSTI vs. TDF | 0.94 | 0.39-2.28 | 0.44 |
| NNRTI vs. TDF | 0.27 | 0.07-0.93 | 0.02 |
| Abbreviations: ART (Antiretroviral Therapy); BMI (Body Mass Index); BMD (Bone Mineral Density); CDC-C (Centres for Disease Control and Prevention – Category C (AIDS CrI (Credible Interval); INSTI (Integrase Strand Transfer Inhibitor); NRTI (Nucleoside Reverse Transcriptase Inhibitor); NNRTI (Non-nucleoside Reverse Transcriptase Inhibitor); OR (Odds Ratio); PTH (Parathyroid Hormone); PI (Protease Inhibitor); TAF (Tenofovir Alafenamide). TDF (Tenofovir Disoproxil); *Posterior probability OR >1 indicates covariate is associated with BMD below -1. | | | |

| **Supplementary table 2. Association of TAF vs. non-TAF/TDF with bone mineral density (BMD) accrual** | | | |
| --- | --- | --- | --- |
| **Covariate** | **Estimated coefficient** | **95% CrI** | **Posterior probability**  **coefficient <0*** |
| TAF vs. non-TAF/TDF | -0.05 | -0.58-0.44 | 0.58 |
| Abbreviations: CrI (Credible Interval); TAF (Tenofovir Alafenamide). TDF (Tenofovir Disoproxil). *Posterior probability co-efficient <0 indicates TAF is associated with a decrease in BMD over the follow up period. | | | |

**Supplementary table 3. Changes to bone health markers over the study period.**

|  |  | **Empirical** | | | **Model** | | |
| --- | --- | --- | --- | --- | --- | --- | --- |
| **Covariate** | **Age group** | **Baseline** | **Follow-up** | **Mean change** | **Mean change**  **(95% CrI)** | **Posterior probability of mean change >0 (increase)** | **Posterior probability of mean change <0 (decrease)** |
| 25-hydroxyvitamin D (nmol/L) | 15-29 | 36.60 | 46.70 | 10.10 | 7.72 (2.29,13.04) | 0.997* | 0.003 |
|  | 20-24 | 37.88 | 49.19 | 11.30 | 7.74 (2.37,13.15) | 0.996* | 0.004 |
| PTH (pmol/L) | 15-29 | 5.71 | 6.11 | 0.40 | 0.21 (-2.25,2.69) | 0.565 | 0.436 |
|  | 20-24 | 9.30 | 6.23 | -3.08 | -2.4 (-4.99,0.01) | 0.026 | 0.975 |
| P1NP (μg/L) | 15-29 | 164.31 | 98.50 | -65.81 | -44.33 (-16.38,-8.6) | 0.003 | 0.997* |
|  | 20-24 | 94.12 | 68.16 | -25.96 | -17.48 (-46.2,9.04) | 0.095 | 0.905 |
| NTX (BCE/mmol creatinine) | 15-29 | 110.05 | 82.56 | -27.49 | -12.03 (-37.22,4.87) | 0.516 | 0.484 |
|  | 20-24 | 55.34 | 58.34 | 3.00 | -0.5 (-16.71,19.47) | 0.514 | 0.486 |
| Abbreviations: BCE (Bone Collagen Equivalents); CrI (Credible Interval); PTH (Parathyroid Hormone); P1NP (procollagen type 1 N-terminal propeptide); NTX (N-terminal telopeptide)  * Significant increase/decrease from baseline (close to 1 statistically significant). | | | | | | | |
